# Chemometric QSAR Modeling and *In Silico* Design of Antioxidant NO Donor Phenols

**DOI:** 10.3797/scipharm.1011-02

**Published:** 2010-12-02

**Authors:** Indrani Mitra, Achintya Saha, Kunal Roy

**Affiliations:** 1 Drug Theoretics and Cheminformatics Lab, Division of Medicinal and Pharmaceutical Chemistry, Department of Pharmaceutical Technology, Jadavpur University, Kolkata 700 032, India; 2 Department of Chemical Technology, University College of Science and Technology, University of Calcutta, 92, A. P. C. Road, Kolkata 700 009, India

**Keywords:** Antioxidants, Chemometric tools, Structure-activity relationships, Phenolic derivatives

## Abstract

An acceleration of free radical formation within human system exacerbates the incidence of several life-threatening diseases. The systemic antioxidants often fall short for neutralizing the free radicals thereby demanding external antioxidant supplementation. Therein arises the need for development of new antioxidants with improved potency. In order to search for efficient antioxidant molecules, the present work deals with quantitative structure-activity relationship (QSAR) studies of a series of antioxidants belonging to the class of phenolic derivatives bearing NO donor groups. In this study, several QSAR models with appreciable statistical significance have been reported. Models were built using various chemometric tools and validated both internally and externally. These models chiefly infer that presence of substituted aromatic carbons, long chain branched substituents, an oxadiazole-N-oxide ring with an electronegative atom containing group substituted at the 5 position and high degree of methyl substitutions of the parent moiety are conducive to the antioxidant activity profile of these molecules. The novelty of this work is not only that the structural attributes of NO donor phenolic compounds required for potent antioxidant activity have been explored in this study, but new compounds with possible antioxidant activity have also been designed and their antioxidant activity has been predicted *in silico*.

## Introduction

Free radicals (reactive oxygen species) like superoxide and hydroxyl radicals are generated as a result of partial reduction of molecular oxygen [1]. Free radicals are constitutively produced during various metabolic functions of the body. In addition to the lethal actions, they bear several beneficial effects also. The immune system utilizes these free radicals for detection of foreign invaders or damaged tissues that are needed to be eliminated from the human system [2]. However, excessive free radical production resulting from heavy exercise, exposure to environmental pollutants, smoking etc may endanger healthy livelihood through an aggravation of their deleterious effects. Recent research implicates a close association of the free radicals (reactive oxygen species accumulating within the human system) with the etiology and/or progression of a number of diseases as well as aging [3]. Most of the fatal degenerative diseases like Parkinson’s disease [4], atherosclerosis involving cardiovascular damage [5] etc have their origin from the deadly effects of these toxic free radicals. The free radicals are also involved in DNA damage [6], induction of lipid peroxidation in cell membranes and inactivation of membrane-bound enzymes.

The free radical attack to the human system can be controlled to a large extent through utilization of antioxidants. Antioxidants [7] are molecules which can safely interact with free radicals and terminate the chain reaction before vital molecules are damaged. To prevent free radical damage, the body has a defense system of antioxidants. But this endogenous antioxidant supply falls short under conditions of excessive oxidative stress. Although there are several enzyme systems within the body that scavenge free radicals, the principle micronutrient (vitamin) antioxidants are vitamin E, beta-carotene and vitamin C [8]. Epidemiologic observations show lower cancer rates in people whose diets are rich in fruits and vegetables suggesting that such diets rich in antioxidants protect the human system against the development of cancer. Antioxidants are also thought to have a role in slowing the aging process and preventing heart disease and strokes. At the molecular level, the antioxidant mechanism of action can be explained based on the electron–proton transfer theories: (a) hydrogen atom transfer (HAT), (b) single-electron transfer–proton transfer (SET-PT) and (c) sequential proton loss electron transfer (SPLET) [9].

The structural features and properties of a molecule determine its biological activity profile. Quantitative structure-activity relationship (QSAR) is a method of studying a series of molecules of different structures with varying observed properties and attempting to find empirical relationships between structure and property or activity [10]. Starting from the period of Hansch [11], QSAR has been widely used for lead optimization and drug discovery process. This technique has also been used by several researchers for designing of newer antioxidant molecules with improved activity. Rastija et al. [12] modeled antioxidant activity of wine polyphenols using QSAR technique with descriptors calculated from 2D and 3D representation of the molecules and inferred that arrangement of free hydroxyl groups on the flavonoid skeleton, or on the phenolic ring together with the shape, size, mass and steric properties of the molecules bear considerable effects on the activity profile of these molecules. Ray et al. [13] performed QSAR modeling using electrotopological state atom (E-state) parameters in order to determine the antiradical properties of flavonoids as studied in a methanolic solution of DPPH (2,2-diphenyl-1-picrylhydrazil) and the antioxidant activity of flavonoids in a beta-carotene-linoleic acid model system and revealed the importance of the substituent effect and structural changes for optimal antioxidant activity of the flavonoids. In order to determine the key chemical features imparting antioxidant activity to this class of molecules, Mitra et al. [14] performed pharmacophore mapping of arylamino-substituted benzo[*b*]thiophenes as free radical scavengers. Various QSAR models of antioxidant molecules have been recently reviewed [15].

Viewing the immense utility of antioxidants for fighting the array of present day diseases, in the present work, an attempt has been made to develop models capable of assessing the structural attributes of a series of molecules required for exhibiting potent antioxidant activity. A series of phenolic compounds with NO donor functions in the molecular structure having significant antioxidant activity was used for QSAR model development in the present work. Besides internal validation, the models developed were validated externally using compounds not included in the model development process. A comparison of the developed QSAR models with a previously reported model for this class of phenolic derivatives has also been performed in the present work. It may be noted that in the previous QSAR report, lower number of compounds were used for model development than those considered in the present work. Based on the QSAR models developed here, a new series of compounds has been designed and their possible antioxidant activity has been predicted *in silico*. The novelty of this QSAR study is not only that the structural requirements for antioxidant activity have been explored in this work, but the developed models have also been used for design of new molecules with possible potent antioxidant activity.

## Materials and methods

### The dataset

The data used for this analysis has been collected from the reports of Boschi et al. [16], Chegaev et al. [17] and Cena et al. [18]. The dataset comprises of 33 phenolic compounds, most of them bearing the NO donor functions, exhibiting a wide range of antioxidant activity. The antioxidant activities of the compounds were reported to be measured using the TBARS (Thiobarbituric acid reactive substance) assay method. For the present work, the IC_50_ (50% inhibitory concentration) values of the compounds were expressed in millimolar units and converted to negative logarithmic scale (pIC_50_). The observed and calculated/predicted activities of the compounds together with their structures are summarized in Tab. 1.

### Descriptor calculation

The molecular structures of the compounds were drawn using the ACD Lab software [19] and were exported to the Cerius2 software [20] for the calculation of descriptors. Initially, conformational analysis of the molecules was performed using ‘optimal search method’ within the Cerius2 software. This method allows the automatic selection of the best method to generate the lowest-energy conformers for structures in the study table. This selection is done among the three methods (grid scan, random sampling and Boltzmann jump) available for conformer generation in the Cerius2 software. Grid scan [20] method performs a simple systematic search in which each specified torsion angle is varied over a grid of equally spaced values. Random sampling [20] perturbs the starting conformation of a structure by randomly altering values of all variable torsion angles and the Boltzmann jump [20] method involves random change of the torsion angles of a molecule within a specified angle window. The lowest energy conformers were energy minimized using the smart minimizer under open force field and the subsequent charge calculation of the lowest energy conformer was performed using Gasteiger method [20]. Followed by this, descriptors belonging to different categories were calculated using the Descriptor+ module of the Cerius2 software version 4.1 [20] (listed in Tab. 2). The calculated topological indices include descriptors like Wiener, Zagreb, Balaban J, connectivity indices (^0^χ, ^1^χ, ^2^χ, ^3^χ_P_, ^3^χ_C_, ^0^χ^v^, ^1^χ^v^, ^2^χ^v^, ^3^χ^v^_P_,, ^3^χ^v^_C_), kappa shape indices (^1^κ, ^2^κ, ^3^κ, ^1^κ_am_, ^2^κ_am_, ^3^κ_am_) and E-state parameters. Besides these, spatial (Jurs charged partial surface area descriptors and shadow indices), structural, physicochemical and electronic descriptors were also calculated [20]. After excluding those descriptors having variance lower than 0.0001, a total of 86 descriptors were chosen. The values of the significant descriptors for all 33 compounds are given in supplementary materials (Tab. S1). Initially a QSAR model was built based on the entire dataset of 33 compounds. Considering the small size of the dataset, full leave-one-out cross-validation [21] has been performed for the model. This was followed by splitting of the dataset into training and test sets for further validation and determination of the external predictive ability of the derived models.

### Selection of training set

The training set was utilized for the development of the QSAR model while the test set was used for the external validation purpose. The selection of the training and test sets serves as a critical step in the QSAR model development process. The selection of the training set should be such that it captures all the features and characteristics of the whole set of molecules. It should also span the activity range of the entire dataset. For the present work, the selection of the training set was done based on the *k*-means clustering technique. Cluster analysis [22] is a method of arranging objects into groups. It divides objects into groups in such a manner that the degree of association between two objects is maximum if they possess same group and otherwise minimum. There are two types of clustering techniques: (a) hierarchical and (b) non-hierarchical. *k*-Means clustering is one of the best known non-hierarchical clustering techniques [22]. In this method, clustering starts randomly and then cluster means are calculated in the descriptor space. Molecules are reassigned to clusters whose means are closer to the position of the molecules. In the present work, clustering was performed with the standardized descriptor matrix using about 25% of the whole dataset compounds as the test set and the remaining as the training set.

### Chemometric tools

Stepwise multiple linear regression (MLR) technique was used for the QSAR model development using the entire dataset. Stepwise MLR method is based on forward selection and backward elimination techniques for inclusion and rejection of descriptors. The selection of the significant descriptors for developing the model was done according to the ‘stepping criteria’ [23] (F) with F = 4 for inclusion and F = 3.9 for exclusion. The F-value used for inclusion or exclusion of a variable in the stepwise regression process is a test for partial regression coefficient and it is obtained by dividing the difference between reductions of sum of squares with and without the variable being included or excluded with error mean square of the equation [23]. The F-value for inclusion or exclusion of a variable in a MLR equation during stepwise process is square of the t-value of the regression coefficient of the variable being included or excluded.

For the development of the QSAR models using the training set data, two different chemometric tools were employed, viz., GFA (genetic function approximation) and G/PLS (genetic partial least squares). A genetic algorithm (GA) is a search technique [24] employed as a computational tool to find out exact or approximate solutions to optimization and search problems. Genetic function approximation was originally conceived from: (i) genetic algorithm originally developed by Fraser and others (later popularized by Holland) and (ii) Friedman’s multivariate adaptive regression splines (MARS) algorithm. In this technique, an initial population of equations is built by random selection of descriptors followed by cross over between pairs of those equations. The progeny equations thus built are again subjected to cross over and the fitness of the final equations is assessed based on the lack of fit (LOF) value (given by Eq. 1). The model quality improves as the value of LOF diminishes.

Eq. 1. $$\text{LOF}=\frac{\text{LSE}}{{\left(1-\frac{c+d\times p}{m}\right)}^{2}}$$

Here, LSE is the least square error, c is the number of basis functions, d is the smoothing parameter which was set at the default value of 1, p is the number of descriptors and m is the number of observations in the training set. In effect, ‘d’ is the user’s estimate of how much detail in the training data set is worth modeling. Smaller equations are obtained for larger values of ‘d’. Since the GFA technique builds a population of equations, the range of variations in this population gives added information on the quality of fit and importance of the descriptors. GFA builds models not only with linear polynomials but also uses higher-order polynomials, splines and other nonlinear functions.

G/PLS technique [25] is the combination of (i) genetic function approximation and (ii) partial least squares methods. These are valuable analytical tools for QSAR modeling where number of descriptors is more than samples. The variables are selected using the GFA technique and the PLS regression method is used to weigh the relative contributions of the selected variables in the final model. Application of G/PLS allows the construction of larger QSAR equations while avoiding overfitting and eliminating most variables. Moreover the PLS technique takes into consideration a large number of noisy and collinear variables. Additionally, PLS provides a description of the available data using minimum number of adjustable parameters and consequently, maximum precision and stability of regression model is achieved using this technique.

### Model quality

Various statistical parameters are calculated in order to assess the fitness of the developed model. The correlation coefficient, R, measures how closely the observed data tracks the fitted regression line and thus helps to quantify any variation in the calculated data with respect to the observed data. The F statistic, calculated from R^2^ and the number of data points, determines the statistical significance of the regression equation at specified degrees of freedom (df). Other statistical parameters used to test the quality of generated regression equations include the standard error of the estimate (*s*) and adjusted R^2^ (R_a_^2^) [23]. Although the value of R^2^ increases with the addition of descriptors to the developed QSAR model but this may not necessarily indicate that the predictive ability of the model improves. Thus, to check the predictive potential of the developed models, internal and external validation experiments are performed on them.

### Model validation

The QSAR models thus developed were validated using both internal and external validation techniques. In case of internal validation, the predictive ability of the models is judged based on the training set compounds. On the contrary, external validation deals with a new set of compounds which are not included in the QSAR model building process. Hence, the latter technique measures the ability of the model to predict the activity of a new series of compounds.

### Internal validation

This technique involves calculation of cross-validated squared correlation coefficient (Q^2^) (Eq. 2) and predicted residual error of sum of squares (PRESS) [23] based on the observed and predicted activity data of the training set molecules. In the present work, leave-one-out (LOO) cross-validation technique was used for determination of Q^2^. For the calculation of LOO-Q^2^, each of the compounds of the training set is deleted once and models are built with the remaining compounds. The activity of the deleted compound is thus predicted using the model developed. The cycle is repeated until all the compounds are deleted at least once and the predicted activity data obtained for all the training set compounds are used for the calculation of above mentioned internal validation parameters.

Eq. 2. $$Q^{2}=1-\frac{\sum {\left(Y_{\text{obs}(\text{train})}-Y_{\text{pred}(\text{train})}\right)}^{2}}{\sum {\left(Y_{\text{obs}(\text{train})}-{\bar{Y}}_{\text{training}}\right)}^{2}}$$

Here, *Y_obs(train)_* is the observed activity, *Y_pred(train)_* is the predicted activity and *Ȳ* *_training_* is the mean observed activity of the training set compounds. A model is considered to be satisfactory if the value of Q^2^ exceeds the stipulated value of 0.5.

### External validation

The value of Q^2^ signifies the ability of the model to predict the activity of molecules which are very much alike the training set ones. But to determine the predictive potential of the QSAR model for a new set molecules differing in some aspects from the training set ones, external validation is needed to be performed. In this case, the predictive capacity of a model is judged by its application for prediction of activity values of the test set compounds and subsequent calculation of Q^2^_ext_, i.e., predictive R^2^ (R^2^_pred_) [26] value as given by Eq. 3:

Eq. 3. $$Q_{\text{ext}}^{2}=R_{\text{pred}}^{2}=1-\frac{\sum {\left(Y_{\text{obs}(\text{test})}-Y_{\text{pred}(\text{test})}\right)}^{2}}{\sum {\left(Y_{\text{obs}(\text{test})}-{\bar{Y}}_{\text{training}}\right)}^{2}}$$

In the above equation, *Y_obs(test)_* and *Y_pred(test)_* are the observed and predicted activity data of the test set compounds. A value of R^2^_pred_ (given by Eq. 3) greater than the stipulated value of 0.5 reflects efficient prediction for the test set molecules by the developed model.

### Calculation of r_m_^2^ metrics

It can be inferred from Eq. 3 that the value of the external predictive parameter (R^2^_pred_) primarily depends on the mean activity value of the training set compounds and its distance from the activity values of the test set compounds. Now, since the value of R^2^_pred_ is dependent on the sum of squared differences between the observed activity data of the test set compounds and the training set mean, the value of R^2^_pred_ increases as these differences for individual compounds increase. Thus, compounds with a wide range of activity data may exhibit a large value for this parameter, but this may not indicate that the predicted activity values are very close to those observed. In such a case, there remains a considerable difference between these values although they maintain a good overall correlation. Thus, to obviate this error and to better indicate the model predictive ability, the r_m_^2^ metrics [27] with threshold values of 0.5 (Eq. 4) were calculated.

Eq. 4. $$r_{m}^{2}=r^{2}\times (1-\sqrt{\left(r^{2}-r_{0}^{2}\right)})$$

In Eq. 4, r^2^ and r_0_^2^ are the squared correlation coefficient values between the observed and predicted activity data [LOO predicted activity of training set compounds in case of r_m_^2^_(LOO)_ and the predicted activity of the test set compounds in case of r_m_^2^_(test)_] with and without intercept respectively. As the above equation (Eq. 4) suggests, the value of r_m_^2^ depends solely on the observed and predicted activity data of the molecules and hence, any large deviation between these values is well be reflected though the r_m_^2^ parameter. Similarly, based on the predicted activity values of both the training and test sets, values of r_m_^2^_(overall)_ [27] were calculated. The parameter r_m_^2^ has been used by different groups of authors to check external predictability of QSAR models [28].

### Randomization tests

Validation of the developed models was also performed using the randomization or Y-scrambling technique. In this technique, the Y column (activity data) is permuted keeping the remaining X matrix (descriptors) unchanged. Thereafter, models are built based on this scrambled matrix and average squared correlation coefficient of the randomized models (R_r_^2^) was calculated. Two types of randomization was performed in the present work, namely, process and model randomization. In case of process randomization, the entire descriptor matrix was used and scrambling of data was done using the total pool of descriptors at 90% confidence level. This technique ensures the reliability and robustness of the process employed for the development of the QSAR model. In addition to this, model randomization was also performed at 99% confidence level using the descriptors occurring in the respective models in order to verify whether the developed QSAR model was the outcome of a chance correlation or not. Values of R_r_^2^ lower than those of R^2^ for the respective model signify a robust model. However, since no guideline is given as to how much this difference should be, another parameter, R_p_^2^ (threshold value=0.5) [27, 29] was calculated (Eq. 5). This parameter penalizes the model R^2^ for small differences between the values of R^2^ and R_r_^2^. Thus, models having an acceptable value for this parameter (>0.5) are considered to be robust enough and are not obtained merely by chance.

Eq. 5. $$R_{p}^{2}=R^{2}\times \sqrt{\left(R^{2}-R_{r}^{2}\right)}$$

However, in an ideal case, the average value of R^2^ for the randomized models should be zero, i.e. R_r_^2^ should be zero. Consequently, in such a case the value of R_p_^2^ should be equal to the value of R^2^ for the developed QSAR model. Thus, the corrected formula of R_p_^2^ (^c^R_p_^2^) as proposed by Todeschini [29] is given as (Eq. 6):

Eq. 6. $${}^{c}R_{p}^{2}=R\times \sqrt{\left(R^{2}-R_{r}^{2}\right)}$$

### Applicability domain

The domain of applicability constitutes an important concept in QSAR analysis that enables estimation of uncertainty in the prediction of a particular molecule based on its similarity to the compounds used for developing the model [30, 31]. It refers to a chemical space as defined by the molecular descriptors and the modeled response. A QSAR model exhibits reliability in prediction only for molecules lying within this defined chemical space referred to as its applicability domain. Thus, for a dissimilar compound lying outside the domain of applicability, reliable prediction of activity becomes unlikely. Consequently, a QSAR should only be used for making predictions of molecules within the specified domain by interpolation thereby enabling avoidance of any unjustified extrapolation for activity prediction. The need to characterize the model applicability domain is also reflected in the OECD guidelines for QSAR model validation [32, 33]. In the present work, applicability domain of the best model selected according to the r_m_^2^_(overall)_ criterion has been assessed. Since the model has been developed based on the G/PLS technique, the DModX method [25] implemented in the SIMCA software [34] has been utilised for detecting the applicability domain of the developed model. In this technique, the residuals of Y and X are used as diagnostic values for ensuring the quality of the model [25]. The standard deviation (SD) of the X-residuals of the corresponding row of the residual matrix E is proportional to the distance between the data point and the model plane in X-space, often called DModX (distance to the model in X-space). Here, X is the matrix of predictor variables, of size N×K [where, N is number of objects (cases, observations) and k is the index of X-variables (k=1, 2, … K)], Y is the matrix of response variables of size N×M [m is the index of Y-variables (m=1, 2, … M)] and E is the N×K matrix of X-residuals. A DModX value larger than around 2.5 times the overall SD of the X-residuals (corresponding to an F-value of 6.25) indicates that the observation is outside the applicability domain of the model [25].

## Results and discussion

Initially, an attempt was made to develop a QSAR model using stepwise regression applied on the whole dataset. This was followed by division of the dataset into training and test sets. Models were developed based on the training set and the developed models were used for prediction of test set activity. Using two different chemometric techniques (GFA and G/PLS), three types of QSAR models were developed based on different combination of descriptors: (a) models developed with topological, structural and thermodynamic descriptors, (b) models developed with spatial, electronic and thermodynamic descriptors and (c) models developed using combined set of descriptors. All the significant models developed in the present work are summarized in Tab. 3. The critical F values at different degrees of freedom at 98% significance level are given at the end of Tab. 3. The results infer that since the F value for each of the QSAR models developed is higher than the corresponding critical value, all the developed models are statistically significant. However, among all the developed models, models developed with the spatial, electronic and thermodynamic descriptors are of poor statistical quality in comparison to the other two types of models. The GFA models were developed using 5000 iterations considering both linear and spline options. The models thus developed are nonlinear, and the spline terms are expressed as truncated power splines and denoted with angular brackets. E. g. <f(x) - a > is equal to zero if the value of f (x) - a is negative, else it is equal to f(x) - a. The constant ‘a’ is called the knot of the spline. G/PLS was performed with 1000 iterations, scaled variables and with the option of no fixed length of equation. The maximum number of components or latent variables (LVs) fixed for variable selection was 3. These components are the functions of the original descriptors and they encode data as represented by the descriptors. Following the model development step, new compounds were designed *in silico* based on the information available from all the developed models (*vide infra*). The activities of all the newly designed compounds were predicted using all the developed QSAR models and their consensus activity values were reported (Tab. 4).

### Model developed with the whole dataset

Eq. 7. $$\begin{array}{l} pIC_{50}=-0.373+0.302(\pm 0.069)\times S\_\text{aasC}+0.009(\pm 0.001)\times \\ \text{JursTASA}-0.017(\pm 0.006)\times \text{MR}-0.360(\pm 0.088)\times {}^{3}X_{c}^{v}-2.11(\pm 0.608)\times \\ S\_\text{ddsN}-1.80(\pm 0.821)\times \text{JursRPCG}\\ n=33,\;s=0.317,\;R^{2}=0.914,\;R_{a}^{2}=0.894,\;F=46.10(df6,26),\\ \text{PRESS}=4.091,\;Q^{2}=0.866,\;\text{true}\;r_{m\;(\text{LOO})}^{2}=0.578 \end{array}$$

In the above equation, n is the number of compounds used for developing the QSAR model. Standard errors of the regression coefficients are shown in parentheses. Eq. 7 was developed with the entire dataset of molecules using stepwise regression method [23]. A value of internal predictive variance (Q^2^ = 0.866) above the stipulated value of 0.5 for the developed model signifies its predictive ability. The positive coefficients for *S_aasC* and *Jurs TASA* descriptors indicate that the antioxidant activities of these molecules increase with an increase in the values of these descriptors. The *S_aasC* descriptor refers to the summation of E-state values for the 
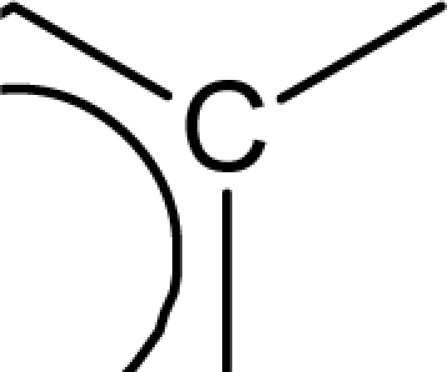
 (aromatic carbon) fragments. *Jurs TASA* (total hydrophobic surface area) is calculated as the sum of solvent-accessible surface areas of atoms with absolute value of partial charges less than 0.2. An increase in the value of *S_aasC* descriptor indicates increase in substitution on the aromatic nucleus while an increase in the value of *Jurs TASA* indicates an increase in surface area with reduced partial charge. Again, negative coefficients for *MR*, *^3^χ^v^_c_*, *S_ddsN* and *Jurs RPCG* descriptors signify that the antioxidant activity of these molecules is inversely proportional to the values of these descriptors. *MR* refers to the molar refractivity of the molecule and gives a measure of the size and volume of the molecule. The parameter, *^3^χ_c_^v^* is a topological descriptor [20] belonging to the category of molecular connectivity indices and defined as the third order cluster index based on valance count. It encodes the number of branch points in a molecule indicating a decrease in branching of the molecule for a decrease in its value. The parameter, *Jurs RPCG* (relative positive charge) is a spatial descriptor obtained by dividing the partial charge of the most positive atom with the total positive charge. *S_ddsN* is a topological descriptor and refers to the summation of E-state value for the nitrogen atom of type 
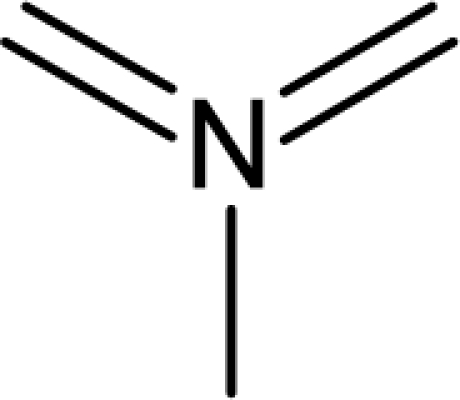
. In this data set, it has referred to the nitrogen of the N-oxide fragment of the oxadiazole ring. It has been observed that the descriptor, *S_ddsN*, assumes a negative value when the 5 position of the oxadiazole ring is substituted with a group containing more electronegative atoms. In such cases, the impact of *S_ddsN* becomes positive on the activity (the coefficient of S-ddSN in Eq. (7) is actually negative).

The opposite signs for the coefficients of *Jurs TASA* and *MR* descriptors infer that although an increase in hydrophobic surface area of the molecules favours their activity, the total volume of the molecule should small enough so that the *MR* descriptor attains a lower value. Thus, the increase in hydrophobic surface area should be up to a specific limit. Interestingly, *MR* assumes a positive coefficient in the absence of the *Jurs TASA* descriptor. Again, according to the order of significance, the descriptors occurring in Eq. (7) can be ranked as: (i) *Jurs TASA*, (ii) *S_aasC*, (iii) *^3^χ_c_^v^*, (iv) *MR*, (v) *S_ddsN* and (vi) *Jurs RPCG*. The weightage of these descriptors once again signifies that the *Jurs TASA* descriptor has a greater impact on the activity profile these molecules compared to the *MR* descriptor. Compound numbers (nos.) **4** and **9** bearing conducive values for all these descriptors exhibit maximum activity. Again compound nos. **16**, **17** and **18** despite having large values for the *MR* and *^3^χ_c_^v^* descriptors exert high range of activity, since these descriptors rank lower in terms of the weightage of the descriptors. Although compound nos. **1**, **10** and **28** satisfy the requirements for most of the descriptors, these compounds exhibit the lowest activity range due to unsatisfactory values of the two most significant descriptors, *Jurs TASA* and *S_aasC*.

It has been argued that in case of a small dataset, considerable amount of information is lost in division of dataset into training and test sets. Alternatively, ‘true r_m_^2^_(LOO)_’ calculated based on the whole dataset may efficiently reflect the predictive potential of a model. Thus, the value of ‘true r_m_^2^_(LOO)_’ [21] (threshold value = 0.5) was also calculated for this dataset. For the calculation of this parameter, each molecule in the dataset was deleted once and the variable selection strategy was applied and a new model was built with the remaining molecules. The activity of the deleted molecule was thereafter predicted using the developed model. The cycle was continued till all the molecules in the dataset were deleted at least once. The activity predicted thus for all the molecules was used for the calculation of this ‘true r_m_^2^_(LOO)_’ parameter. Thus, in the calculation of ‘true r_m_^2^_(LOO)_’ metric, new variables are selected in each cycle based on the leave-one-out technique. Consequently, this parameter reflects the external predictive ability of the model especially in case of such a small dataset where splitting may result in loss of an appreciable amount of chemical information. In this case, statistically significant result was obtained for the ‘true r_m_^2^_(LOO)_’ (0.578) parameter indicating ability of the model to predict the activity of new series of molecules of this class.

### Models developed from training set data

To indicate the external predictivity of developed models, the dataset was further divided into training and test sets. Subsequent models were built based on the training set and were externally validated based on the test set. Three different types of models were built using the training set compounds such as: (i) models developed with topological, structural and thermodynamic descriptors, (ii) models developed with spatial, electronic and thermodynamic descriptors and (iii) models developed with combined set of descriptors. The predictive ability of the models was judged based on the internal and external validation parameters which are summarized in Tab. 3. All the models bear acceptable values of Q^2^ and R^2^_pred_ which are considerably greater than the stipulated value of 0.5. In terms of internal predictivity (Q^2^ = 0.909), model C1 developed with the combined set of descriptors was the better compared to the other ones. But since internal validation alone fails to judge the ability of a model to predict the activity of new series of molecules, values of R^2^_pred_ were also taken into consideration. Thus, in terms of both internal (Q^2^ = 0.814) and external (R^2^_pred_ = 0.917) predictive parameters, model A2a developed with the topological, structural and thermodynamic descriptors shows maximum statistical significance. However, the value of R^2^_pred_ alone fails to judge whether the range of predicted activity data lies within the desired observed activity range. Hence, values of r_m_^2^ metrics were calculated. The r_m_^2^_(overall)_ value determines the degree of proximity between the observed and corresponding predicted activity data for both the training and test set molecules. Thus, in terms of all the three parameters (Q^2^ = 0.877, R^2^_pred_ = 0.884, r_m_^2^_(overall)_ = 0.872), model A4 developed with topological, structural and thermodynamic descriptors exhibits maximum statistical significance. It can be inferred from Tab. 3 that the models developed with spatial, electronic and thermodynamic descriptors were inferior in terms of their predictive ability compared to the remaining ones and hence are not described below.

The repeated occurrence of the E-state descriptors in the developed QSAR models signifies the importance of the various structural fragments for optimal antioxidant activity of these molecules. The *S_aaCH* and *S_aasC* descriptors refer to the summation of E-state values for unsubstituted ( 
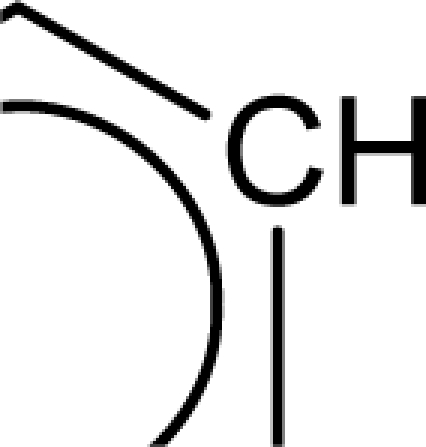
) and substituted ( 
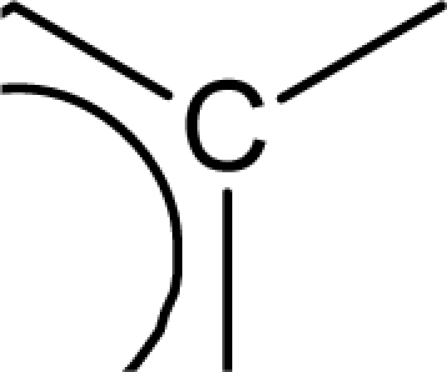
) aromatic carbon fragments respectively while *S_sCH_3_* descriptor refer to the summation of E-state values for the methyl groups (−CH_3_) present within the molecular structures. Thus, presence of these descriptors signifies the influence of these structural fragments for the activity profile of these molecules. The parameter *S_aasC* also appeared in Eq. 7 obtained for the whole dataset. Again repeated occurrence of the *Jurs* descriptors and the various types of connectivity (χ) descriptors indicate that the charged surface area of the molecules as well as their extent of branching influence the antioxidant activity profile of these molecules.

Due to space limitation, only the GFA and G/PLS models (models A2, A2a and A4) obtained using spline option from topological, structural and thermodynamic descriptors are described here.

### GFA model

Eq. 8. $$\begin{array}{l} pIC_{50}=-1.68-0.113(\pm 0.029)\times <SC-3\_P-20>+0.786(\pm 0.164)\times {}^{3}X_{p}\\ -0.637(\pm 0.099)\times <1.79401-S\_s{\text{CH}}_{3}>+0.329(\pm 0.049)\times S\_\text{aasC}\\ n_{\text{training}}=25,s=0.315,\;R^{2}=0.919,\;R_{a}^{2}=0.903,\;F=56.95(df4,20),\text{PRESS}=3.022,\\ Q^{2}=0.877,\;r_{m\;(\text{LOO})}^{2}=0.757,\;n_{\text{test}}=8,\;R_{\text{pred}}^{2}=0.924,\;r_{m\;(\text{test})}^{2}=0.899,\;r_{m\;(\text{overall})}^{2}=0.777 \end{array}$$

The acceptable values of the internal (Q^2^ = 0.877) and external (R^2^_pred_ = 0.924) predictive parameters reflect the predictability of the developed model. Moreover, statistically significant results for all the r_m_^2^ metrics indicate that the predicted activity values of all the compounds are close to the corresponding observed activity data. Although the model exhibits high predictive ability, there exists significant intercorrelation between two descriptors namely, SC-3_P (number of third-order subgraphs in the molecular graph) and ^3^χ_p_ (molecular connectivity index). Intercorrelation matrix (Tab. 5) for all the descriptors appearing in Eq. 8 signifies that there may exist a parabolic relationship between the activity values and these descriptors. Thus, to better express the parabolic behaviour of the developed model, the SC-3_P descriptor was replaced with a second order function of the ^3^χ_p_ descriptor.

Eq. 9. $$\begin{array}{l} pIC_{50}=-0.448+0.497(\pm 0.147)\times {}^{3}X_{\text{p}}-0.0225(\pm 0.009)\times {\left({}^{3}X_{\text{p}}\right)}^{2}\\ +0.300(\pm 0.110)\times S\_\text{aasC}-0.539(\pm 0.057)\times <1.79401-S\_sCH_{3}>\\ n_{\text{training}}=25,\;s=0.369,\;R^{2}=0.889,\;R_{a}^{2}=0.867,\;F=40.17(df4,20),\\ \text{PRESS}=4.557,\;Q^{2}=0.814,\;r_{m\;(\text{LOO})}^{2}=0.677,\;n_{\text{test}}=8,\\ R_{\text{pred}}^{2}=0.917,\;r_{m\;(\text{test})}^{2}=0.818,\;r_{m\;(\text{overall})}^{2}=0.711 \end{array}$$

Eq. 8 is thus modified to Eq. 9 in order to express the parabolic relationship of the developed QSAR model with respect to ^3^χ_p_ descriptor. ^3^χ_p_ [20] is the weighted count of four atom (three-bond) fragments and it reflects the degree of branching at each of the four atoms in the fragment. In the above equation, a positive coefficient of the ^3^χ_p_ descriptor signifies that the antioxidant activity of these molecules increases with an increase in the value of this descriptor. Thus, it can be inferred that an increase in the degree of branching in these molecules favours their antioxidant activity profile. Maximum antioxidant activity profile of compound nos. **9**, **16** and **18** can be explained by their large ^3^χ_p_ descriptor values while the reduced activity of compound nos. **1**, **6**, **10** and **28**, may be attributed to the small values of ^3^χ_p_ descriptor. The optimum value of ^3^χ_p_ for this series of compounds is 11.044 which approximately matches with the value of this descriptor for the most active compound (compound no. **18**). This in turn explains the parabolic relationship between activity and ^3^χ_p_ descriptor. The *S_aasC* descriptor refers to the summation of E-state values for the 
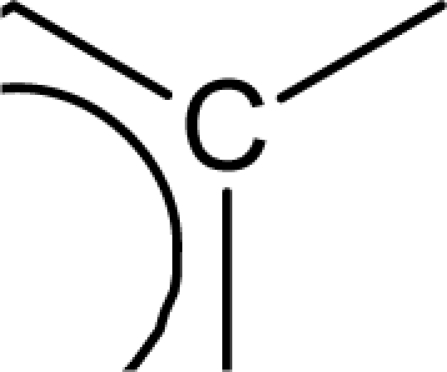
 (aromatic carbon) fragments. Since the *S_aasC* descriptor bears a positive coefficient, an increase in its value leads to an improvement in the antioxidant activity profile of these compounds as observed in case of compound nos. **9**, **16**, **17** and **18**. Thus, as the number of such fragments increase, an increase in the activity data of these molecules is observed. The *S_sCH_3_* descriptor refers to the summation of E-state values for the −CH_3_ (methyl group) fragments. In the above equation, a negative coefficient of the spline term with this descriptor indicates that for any value of this descriptor greater than 1.79401, the spline term<1.79401–*S _ sCH*_3_ >, exerts zero contribution and the compounds show an improvement in activity data as exemplified in compound nos. **9**, **16**, **17** and **18**. On the contrary, compound nos. **6**, **10**, **28** and **33** with zero values of the *S_sCH_3_* descriptor exhibit lowest activity range. Thus it can be suggested that presence of methyl substituents favours the antioxidant activity profile of these molecules.

### G/PLS model

A statistically significant QSAR model was also obtained using the G/PLS technique together with the spline option.

Eq. 10. $$\begin{array}{l} pC=1.158-0.429\times <6.68154-{}^{1}X>+0.108\times^{3}X_{p}\\ -0.525\times <1.98556-S\_{\text{sCH}}_{3}>+0.288\times S\_\text{aasC}\\ n_{\text{training}}=25,\;\text{LVs}=2,\;s=0.323,\;R^{2}=0.905,\;R_{a}^{2}=0.897,\;F=106.44(df2,22),\\ \text{PRESS}=3.023,\;Q^{2}=0.877,\;r_{m\;(\text{LOO})}^{2}=0.870,\;n_{\text{test}}=8,\;R_{\text{pred}}^{2}=0.884,\\ r_{m\;(\text{test})}^{2}=0.812,\;r_{m\;(\text{overall})}^{2}=0.872 \end{array}$$

The predictive power of the developed model is reflected through the statistically significant values of the internal (Q^2^ = 0.877) and external (R^2^_pred_ = 0.884) validation parameters as well as the r_m_^2^ metrics. Among all the developed equations, this equation gives a maximum value of the r_m_^2^_(overall)_ (0.872) parameter indicating that the predicted activity values of all the dataset compounds are in very close proximity to the corresponding observed data. These results signify that the model can be efficiently used for activity prediction of new compounds of this class. The predicted activity values (LOO predicted values for the training set) according to Eq. (10) are given in Tab. 1. According to the order of significance, the descriptors occurring in Eq. (10) can be arranged as: (i) *<1.98556-S_sCH_3_>*, (ii) *S_aasC*, (iii) ^3^χ_p_ and (iv) <6.68154-^1^χ>. Similar to the previous equation (Eq. 9), ^3^χ_p_ and *S_aasC* descriptors bear positive coefficients indicating that the antioxidant activity increases with an increase in the values of these descriptors. Consequently, this observation infers that a high degree of branching (as indicated by high values of ^3^χ_p_) and large number of 
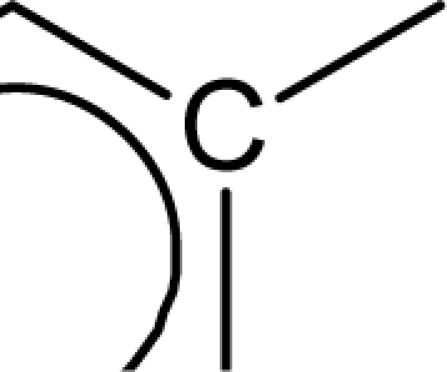
 fragments present within the molecular structure of these NO donor phenolic compounds favour their antioxidant activity profile.

Again, negative coefficients of the spline terms with the *S_sCH_3_* and *^1^χ* imply that for zero values of these spline terms, the compounds show high activity range. This, in turn, suggests that values of *S_sCH_3_* descriptor greater than 1.98556 and that of *^1^χ* descriptor greater than 6.68154 account for zero contribution of the spline function and hence explain the high activity range for compounds with such values. This is because a negative value of a spline term indicates zero contribution of the corresponding spline term. *^1^χ* [20] is a topological descriptor referring to simple connectivity index obtained by one bond dissection of the molecule. Since the descriptor encodes the number of non-hydrogen atoms in a molecule, values of this descriptor reflect the size and volume of the molecule along with the degree of branching. Thus the results suggest that an increase in the number of methyl substitution (−CH_3_ fragment) as well as an increase in the volume and/or branching of the molecule favours their antioxidant activity. This increase in size can be well correlated with the observation of Eq. 7, which infers that increase in total hydrophobic surface area of these molecules up to a definite limit favors their antioxidant activity profile. Compound nos. **9**, **16**, **17** and **18** fulfilling all these necessary structural requirements exert maximum activity range. In case of compound nos. **6**, **10** and **28**, although the <6.68154-^1^χ> descriptor attains zero value, the other highly significant descriptors do not meet the necessary criteria and subsequently, results in a lowering of their activity profile. Similarly, compound no. **33** shows reduced activity despite having zero value for the <6.68154-^1^χ> descriptor and a large value of the ^3^χ_p_ descriptor due to a lower weightage of these descriptors compared to the others.

### Major observations from other models

The remaining equations which are not described in detail here also reveal some interesting structure-activity relationships for optimum antioxidant activity of these molecules. The *S_dsN* descriptor appearing in model A3 refers to the summation of E-state values for the −N= fragment of the pyrazolone ring. The positive coefficient of this term indicates that the presence of this fragment in the molecular structure favours the activity range of these compounds. However, being a descriptor of less relative importance, it does not significantly contribute to the activity profile of these compounds. Again, both models C2 and C4 bear the *Rad of Gyra* descriptor (abbreviation for radius of gyration, which is a size descriptor denoting the distribution of atomic masses in a molecule and measures of molecular compactness and symmetry for long-chain molecules) inferring that long chain unsymmetrical substitution of the parent molecule leads to an increase in their activity profile. Besides these, most of the models signify that an increase in the activity profile of these molecules is achieved with an increase in the degree of methylation and substituted aromatic carbon fragments in their molecular structure.

### Results of validation based on randomization tests

Further validation of the developed models was done using the randomization technique in order to check the robustness of the genetic QSAR models. Process randomization was performed using the whole descriptor matrix to assess the fitness of the process employed for the development of the QSAR models. Besides this, model randomization was also performed in order to determine whether the model was developed by chance or not. Based on the randomized data, values of R_r_^2^ were calculated. For all the developed models, the values of R_r_^2^ were much lower than that of the model R^2^ implying the robustness of the developed model. However, since no guideline is given as to how much this difference should be in order to obtain a robust QSAR model, values of ^c^R_p_^2^ were computed [29] (Tab. 6). Models having ^c^R_p_^2^ values greater than 0.5 are considered to be statistically robust. The ^c^R_p_^2^ values of most of the models obtained for the process randomization technique exceed the threshold value of 0.5. The ^c^R_p_^2^ values for all the models described above are well above the stipulated value with model A4 (0.899) and model C4 (0.913) exhibiting maximum values. This indicates that the models developed are not merely the outcome of chance.

### Design of new compounds

**Fig. 1.**
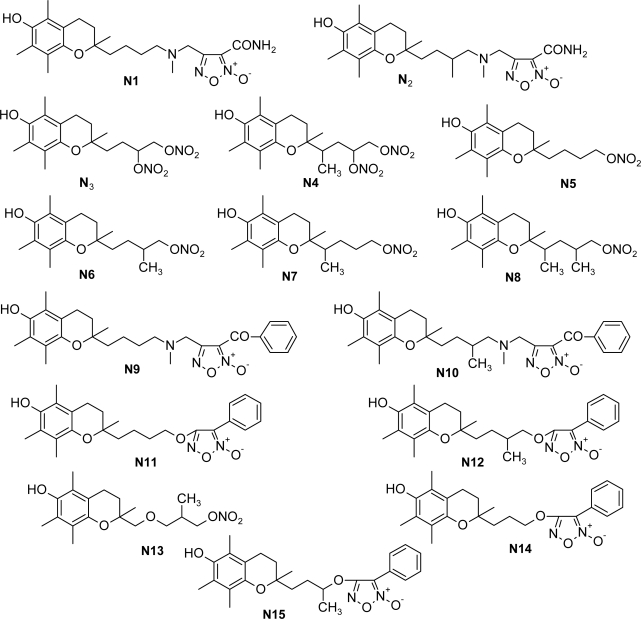
Structures of the designed compounds

The statistically significant QSAR models developed above thus determine the required structural attributes for maximum antioxidant activity. The equations primarily suggest that the presence of substituted aromatic carbon within the molecular structure together with extensive methyl substituent favours the antioxidant activity profile of this series of molecules. Additionally charged surface area and polar surface are of the molecules also play significant role in determining the potency of these molecules indicating that an increase in surface area and volume of the molecules up to a specific limit favours their activity profile. Besides these, charged positive and charged polar surface areas play an important role for activity prediction of these molecules. Moreover, long chain compounds with reduced symmetry and an optimum volume may favour the activity profile of these compounds. An extensive occurrence of the connectivity descriptors in the above equations signify that proper branching of the molecules essential for potent antioxidant activity of these molecules. Again, the developed QSAR models also suggest that an oxadiazole-N-oxide ring substituted with an electronegative atom containing group at the 5 position may exert a positive impact on the activity of the molecules. Based on these structural attributes, 15 new molecules were designed and their activity was predicted using all the developed models. With the predicted activity obtained for all the developed models, a consensus for predicted activity was computed and it was observed that all the compounds exhibited potent activity profile which was in close proximity to that of the highly active compounds of the present dataset. All the 15 designed compounds and their *in silico* predicted activity data are listed in Fig. 1 and Tab. 4.

### Test for applicability domain

**Fig 2.**
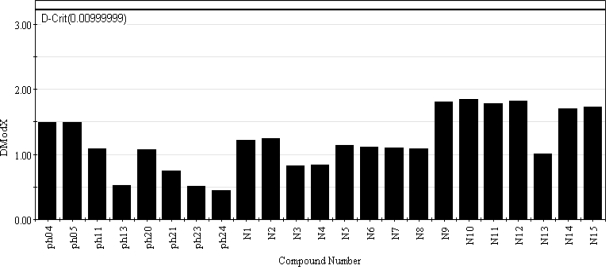
Bar diagram showing the DModX values of the 8 test set compounds and the 15 designed compounds calculated at 99% significance level with the thick horizontal line signifying the critical DModX value (3.225) for Eq. (10).

According to the r_m_^2^_(overall)_ criterion, Eq. (10) is the best model among all the developed models. The applicability domain Eq. 10 (G/PLS model) was checked based on the DModX [25] approach. A bar diagram for the DModX values of the 8 test set compounds as well as the designed compounds for Eq. (10) is shown in Fig. 2. The DModX values thus obtained for all the test compounds as well as the 15 newly designed compounds are below the critical value of 3.225 calculated at the 99% significance level. So, none of the compounds are outside the applicability domain of Eq. (10) and predictions for all the compounds are acceptable with confidence. Moreover, acceptable DModX values for the designed compounds indicate that predictions for antioxidant activity for these compounds according to Eq. (10) are reliable.

The best model [Eq. (10)] thus built obeys the 5 guidelines for acceptability of QSAR models laid by the Organisation for Economic Co-operation and Development (OECD) [35]: (i) the model has been built based on an unambiguous algorithm; (ii) a definite response, viz., antioxidant activity using the TBARS (Thiobarbituric acid reactive substance) assay method has been modeled in the present work; (iii) the molecules predicted using the developed model are rightly located within the model applicability domain; (iv) goodness of fit, robustness and predictivity of the developed models have been appropriately checked using different validation measures and (v) the model provides a suitable mechanistic interpretation for assessing the necessary structural attributes of the molecules for exhibiting optimum response. Hence, the developed model can be satisfactorily used from the regulatory point of view.

### Comparison with previously reported work on NO donor phenols

Previously, structure–antioxidant activity relationships for a series of NO-donor phenols have been reported by Tosco et al. [36]. 17 out of the 33 NO donor phenols used in the present work were modeled by Tosco et al. based on their partition coefficient and bond dissociation enthalpy values. Tosco et al. reported structure-activity relationships of these molecules purely based on their internal validation parameters. They developed a bilinear model with 17 compounds and obtained a Q^2^ of 0.94. However, as Tosco et al. used limited number of compounds for QSAR model development and did not perform external validation, a direct comparison of these models with those developed by us is not possible. Unlike the present work, they did not report any data regarding external validation and randomization of the dataset.

## Overview and conclusion

In the present work, QSAR models were built using a dataset (n=33) comprising phenolic derivatives but chiefly constituting compounds with the NO donor functions. For the development of the QSAR models different statistical tools and software were employed. The major chemometric tools used for the present work include the GFA and G/PLS techniques. Initially QSAR model was developed using the entire dataset using stepwise regression and value of “true r_m_^2^_(LOO)_” was calculated in order to determine the predictive ability of the dataset. In order to determine the external predictive ability of models, the dataset was divided into training and test sets using the *k*-means clustering technique and external validation was done based on the activity prediction of the test set compounds. Eq. 9 (0.917) with maximum value of the R^2^_pred_ parameter indicates significant ability of the developed model to predict the activity of new compounds belonging to this series of phenolic derivatives. Besides these, the r_m_^2^ metrics were also calculated to determine the distance of the predicted activity data from the corresponding observed ones. A high value of r_m_^2^_(overall)_ for Eq. 10 (0.872) implies that the activity data predicted for the test set compounds using the model satisfies the desired range of observed activity data. To check the reproducibility of the developed models, validation was done using both process and model randomization techniques. The results of model randomization test reveal that the ^c^R_p_^2^ values [37] for all the models exceed the stipulated value of 0.5. Maximum ^c^R_p_^2^ values for model A4 and model C4 infer that the developed models are sufficiently robust and not the outcome of mere chance.

Analysis of the QSAR models developed in the present work reveal the structural requirements of these molecules for exhibiting maximum antioxidant activity. The repeated occurrence of the *S_aasC* and *S_CH_3_* descriptors in different models signifies that the presence of the 
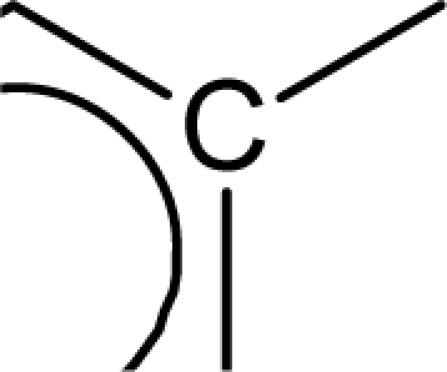
 fragment and methyl substituents within the molecular structure of these phenolic derivatives is conducive to the antioxidant activity profile of these compounds. The presence of an aromatic carbon without a substitution ( 
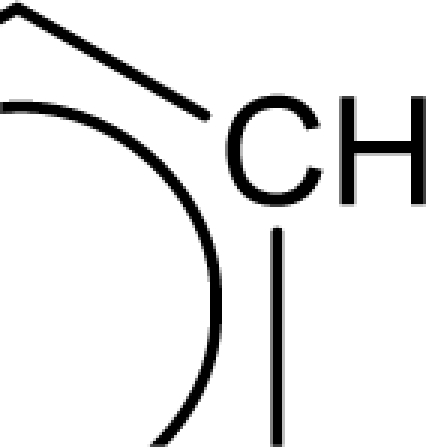
) hinders the activity profile of these compounds. The presence of an oxadiazole-N-oxide ring with an electronegative atom containing group substituted at the 5 position are conducive for the antioxidant activity of these compounds. Besides these, increase in the positively charged surface area and the volume of the molecules favours the antioxidant activity profile of these compounds. Long chain branched substituents lacking symmetry about the centre of mass of the molecule exhibit improved antioxidant activity. Based on this structural information, 15 new compounds were designed and their activity was predicted using the QSAR models developed in the present work. Since the qualities of the models are good and the observed and predicted activity values of the test set compounds are in good agreement, we can presume that the designed compounds may show potent experimental antioxidant activity as also predicted by the developed models. Thus, the statistically significant QSAR models developed in the present work can be satisfactorily used for activity prediction of new series of molecules of this class. Moreover, the compounds designed in the present work can be utilized further for experimental work.

## Supporting Information



## Figures and Tables

**Tab. 1.** Molecular structure together with the observed and predicted activity data of the 33 phenolic derivatives. 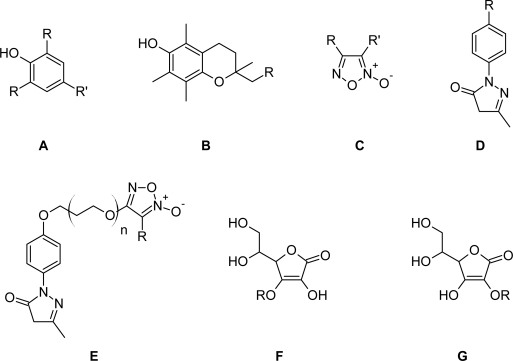

**Compd. No. / Structure**		**R**	**R' / n**	**pIC_50_** **[log(1/IC_50_)] [16–18]**	**Activity predicted^a^**	**Activity predicted^b^**
**1**	A	H	CH_3_	0.538	−0.405	0.639
**2**	A	OCH_3_	CH_3_	1.745	1.760	1.700
**3**	A	*t*-Bu	CH_3_	2.770	3.039	2.732
4*	B	H	–	3.770	3.475	3.451
5*	C	OEt	SO_2_Ph	0.959	1.403	1.367
**6**	A	H	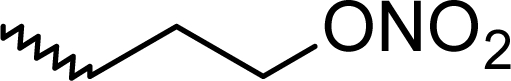	0.845	1.275	0.932
**7**	A	OCH_3_	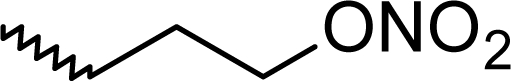	2.229	2.434	2.180
**8**	A	*t*-Bu	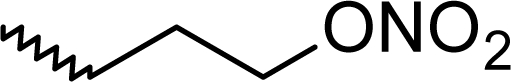	2.699	2.583	2.817
**9**	B	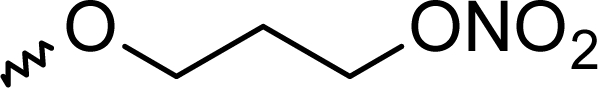	–	3.824	4.064	3.452
**10**	A	H	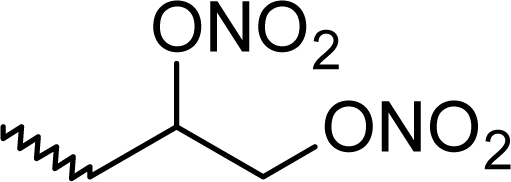	0.733	0.780	0.910
11*	A	OCH_3_	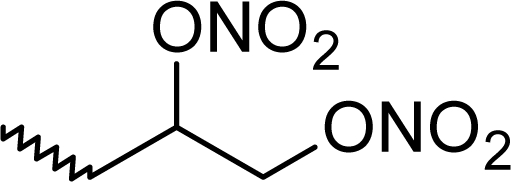	2.268	2.031	2.057
**12**	A	*t*-Bu	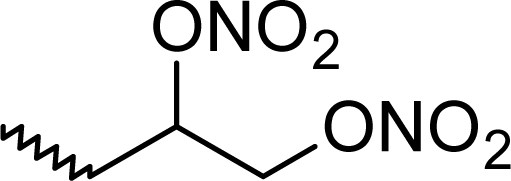	2.585	2.408	2.688
13*	A	H	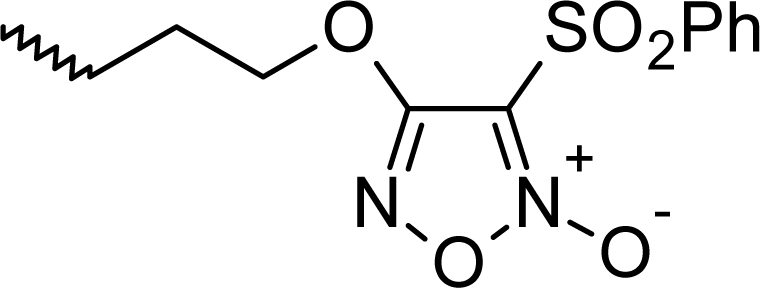	1.328	1.277	1.132
**14**	A	OCH_3_	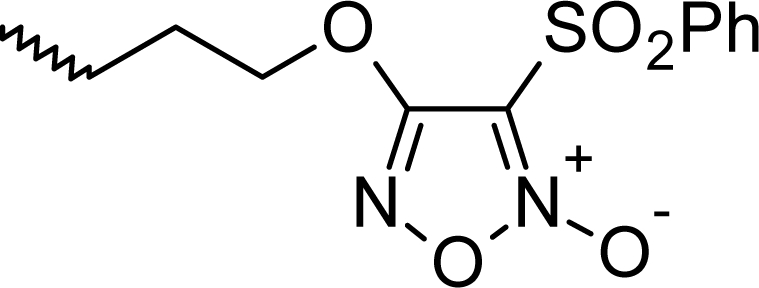	2.469	2.676	2.310
**15**	A	*t*-Bu	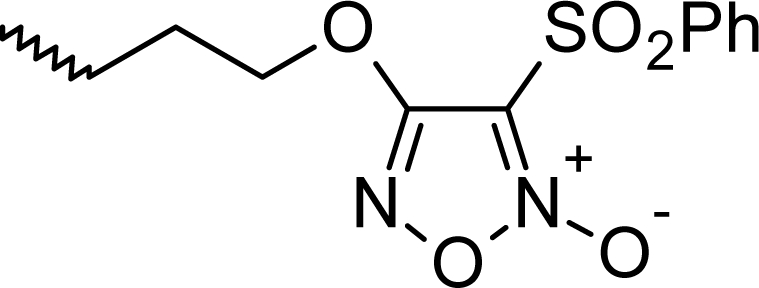	2.699	2.490	3.039
**16**	B	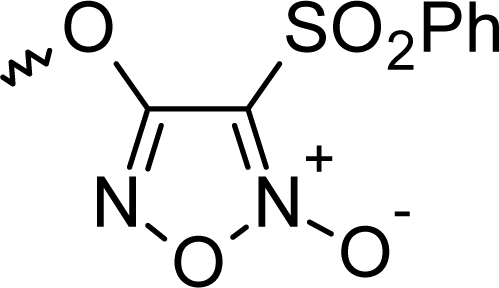	–	3.310	3.649	3.448
**17**	A	*t*-Bu	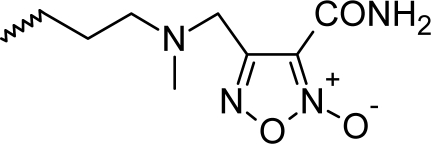	2.921	2.519	3.218
**18**	B	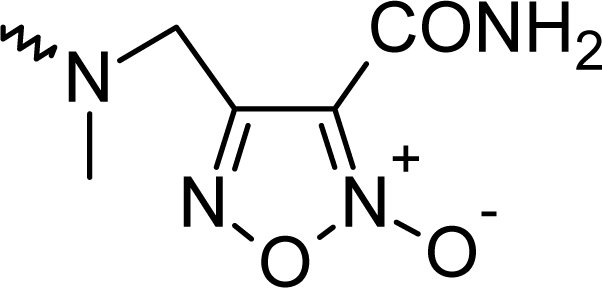	–	3.854	4.063	3.626
**19**	D	H	–	1.770	2.021	1.626
20*	D	OCH_3_	–	1.699	2.006	2.170
21*	D	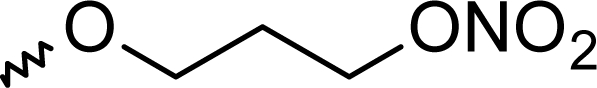	–	2.469	2.254	2.171
**22**	D	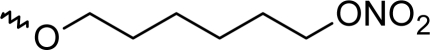	–	2.678	2.964	2.280
23*	D	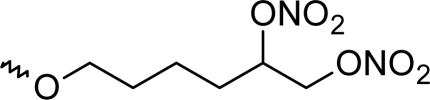	–	2.538	2.532	2.348
24*	E	Ph	1	2.886	3.023	2.936
**25**	E	SO_2_Ph	1	2.420	2.617	2.341
**26**	E	CONH_2_	0	2.102	1.810	2.169
**27**	E	CN	0	2.237	2.004	2.254
**28**	F	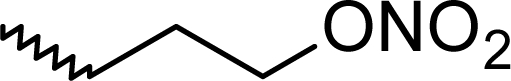	0.343	−0.128	0.829	
**29**	F	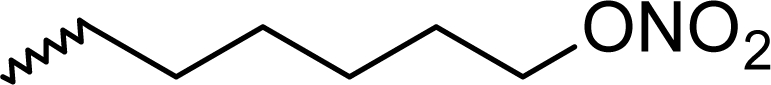	1.097	1.327	0.803	
**30**	F	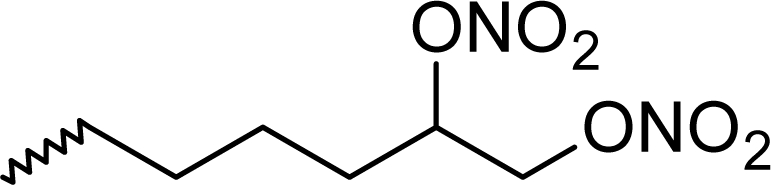	1.553	2.115	0.869	
**31**	F	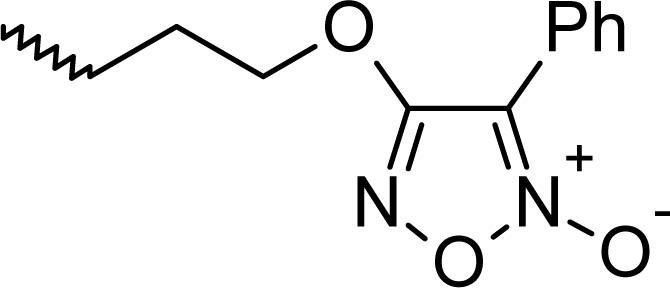	1.770	2.074	1.361	
**32**	F	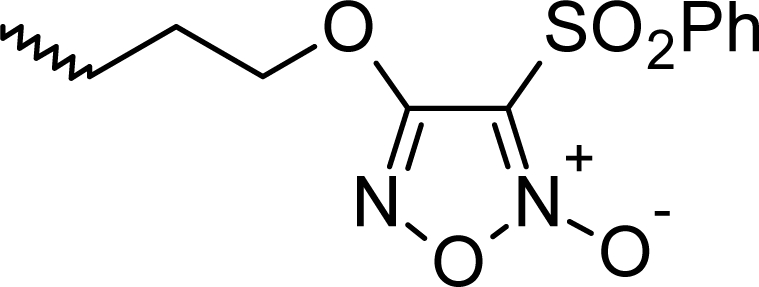	1.097	1.422	0.865	
**33**	G	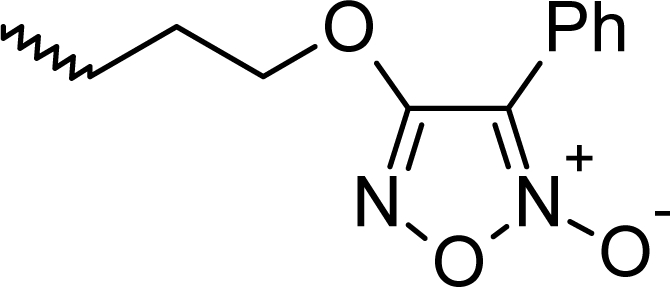	0.407	−0.914	1.548	

* Compounds selected as test set based on *k*-means clustering;

a,b Activity predicted (LOO predicted for the training set) based on Eqs. 8 and 10, respectively.

**Tab. 2.** List of descriptors used for present work.

**Category of descriptors**	**Descriptor type**
Topological indices	Wiener, Zagreb, Balaban, connectivity indices, kappa shape indices, E-state parameters
Structural	Hbond acceptor, Hbond donor, Rotlbonds, Chiral centers
Thermodynamic	LogP, AlogP, AlogP98, Molar refractivity
Spatial	Jurs descriptors, Shadow indices, Radius of Gyration, Molecular surface area, Density, Principal moment of inertia, Molecular volume.
Electronic	Dipole moment, HOMO (Highest occupied molecular orbital energy), LUMO (Lowest unoccupied molecular orbital energy), Superdelocalizability (Sr).

**Tab. 3.** Comparison of the statistical quality of the various QSAR models developed in the present work.

**Using topological, structural and thermodynamic descriptors**										

**Mod.**	**Stat.**	**Eq.**	**Descriptors**	**LVs**	**n_train._**	**s**	**R^2^**	**R^2^_a_**	F*	**PRESS**

A1	GFA-linear	–	SC-0, S_aaCH, S_dssC, S_dO <SC-3_P-20>,	–	25	0.369	0.889	0.867	40.07	4.756
A2	GFA-spline	8	^3^χ_p_, <1.79401-S_sCH_3_>, S_aasC^3^χ_p_, (^3^χ_p_)^2^,	–	25	0.315	0.919	0.903	56.95	3.021
A2a	GFA-spline	9	<1.79401-S_sCH_3_>, S_aasC S_aaCH,	–	25	0.369	0.889	0.867	40.17	4.557
A3	G/PLS-linear	–	S_aasC, S_dsN, S_sOH, MolRef <6.68154-^1^χ >,	2	25	0.401	0.856	0.843	65.48	5.150
A4	G/PLS-spline	10	^3^χ_p_, <1.98556-S_sCH3>, S_aasC	2	25	0.323	0.906	0.897	106.44	3.022

**Mod.**	**Stat.**	**Eq.**	**Descriptors**	**Q^2^**	**r_m_^2^_(LOO)_**	**n_test_**	**R^2^_pred_**	**r_m_^2^_(test)_**	**r_m_^2^_(overall)_**	

A1	GFA-linear	–	SC-0, S_aaCH, S_dssC, S_dO <SC-3_P-20>,	0.806	0.676	8	0.859	0.839	0.685	
A2	GFA-spline	8	^3^χ_p_, <1.79401-S_sCH_3_>, S_aasC ^3^χ_p_, (^3^χ_p_)^2^,	0.877	0.757	8	0.924	0.899	0.777	
A2a	GFA-spline	9	<1.79401-S_sCH_3_>, S_aasC S_aaCH,	0.814	0.677	8	0.917	0.818	0.711	
A3	G/PLS-linear	–	S_aasC, S_dsN, S_sOH, MolRef <6.68154-^1^χ >,	0.790	0.771	8	0.879	0.887	0.790	
A4	G/PLS-spline	10	^3^χ_p_, <1.98556-S_sCH3>, S_aasC	0.877	0.870	8	0.884	0.812	0.872	

**Using spatial, electronic, and thermodynamic descriptors**										

**Mod.**	**Stat.**		**Descriptors**	**LVs**	**n_train._**	**s**	**R^2^**	**R^2^_a_**	F*	**PRESS**

B1	GFA-linear		MR, Jurs-TASA	–	25	0.443	0.824	0.808	51.55	5.271
B2	G/PLS-linear		MR, Jurs-SASA, Jurs-PPSA-3, Jurs-TASA	3	25	0.434	0.839	0.816	36.41	5.516
B3	G/PLS-spline		<55.0428-MR>, Jurs-PNSA-2, <121.354-Jurs-WNSA-1>, Jurs-WPSA-3, <472.813-Jurs-TASA>	3	25	0.397	0.865	0.846	44.88	4.901

**Mod.**	**Stat.**		**Descriptors**	**Q^2^**	**r_m_^2^_(LOO)_**	**n_test_**	**R^2^_pred_**	**r_m_^2^_(test)_**	**r_m_^2^_(overall)_**	

B1	GFA-linear		MR, Jurs-TASA	0.785	0.645	8	0.754	0.683	0.639	
B2	G/PLS-linear		MR, Jurs-SASA, Jurs-PPSA-3, Jurs-TASA <55.0428-MR>, Jurs-PNSA-	0.775	0.754	8	0.773	0.661	0.775	
B3	G/PLS-2, spline		<121.354-Jurs-WNSA-1>, Jurs-WPSA-3, <472.813-Jurs-TASA>	0.800	0.787	8	0.678	0.525	0.761	

**Using combined descriptors**										

**Mod.**	**Stat.**		**Descriptors**	**LVs**	**n_train._**	**s**	**R^2^**	**R^2^_a_**	F*	**PRESS**

C1	GFA-linear		*^3^χ_c_^v^*, Zagreb, S_aaCH, Jurs-RPSA	–	25	0.284	0.935	0.921	71.35	2.237
C2	GFA-spline		<4.19273-*^1^χ^v^* >, S_aasC, <1.83917-S_sCH_3_>, RadOfGyration	–	25	0.304	0.925	0.910	61.31	2.756
C3	G/PLS-linear		*^0^χ^v^*, S_aaCH, S_aasC, S_sOH, Jurs-TASA, HOMO <1.78363-S_sCH_3_>,	2	25	0.362	0.883	0.872	82.95	5.205
C4	G/PLS-spline		<S_aasC-1.50199>, <5.22431-RadOfGyration>, <133.005-Jurs-WPSA-2>	1	25	0.294	0.919	0.915	260.7	3.064

**Mod.**	**Stat.**		**Descriptors**	**Q^2^**	**r_m_^2^_(LOO)_**	**n_test_**	**R^2^_pred_**	**r_m_^2^_(test)_**	**r_m_^2^_(overall)_**	

C1	GFA-linear		*^3^χ_c_^v^*, Zagreb, S_aaCH, Jurs-RPSA	0.909	0.808	8	0.894	0.834	0.822	
C2	GFA-spline		<4.19273-*^1^χ^v^* >, S_aasC, <1.83917-S_sCH_3_>, RadOfGyration	0.888	0.768	8	0.892	0.826	0.791	
C3	G/PLS-linear		*^0^χ^v^*, S_aaCH, S_aasC, S_sOH, Jurs-TASA, HOMO <1.78363-S_sCH_3_>,	0.788	0.758	8	0.880	0.800	0.785	
C4	G/PLS-spline		<S_aasC-1.50199>, <5.22431-RadOfGyration>,<133.005-Jurs-WPSA-2>	0.875	0.848	8	0.800	0.737	0.829	

Critical values of F distribution (two-tailed) at 98% significance level: F_4, 20_ = 4.431, F_2, 22_ = 5.719, F_3, 21_= 4.874, F_1, 23_ = 7.881

**Tab. 4.** Activity predicted for the newly designed compounds based on the 11 QSAR models developed in the present work.

**Cpd. No.**	**Model A1**	**Model A2**	**Model A3**	**Model A4**	**Model B1**	**Model B2**	**Model B3**	**Model C1**	**Model C2**	**Model C3**	**Model C4**	APA*
N1	4.085	9.362	4.281	3.915	3.213	2.697	3.282	4.900	7.824	3.851	8.831	5.113
N2	4.245	10.777	4.368	3.945	3.818	3.170	3.055	4.497	9.414	4.117	10.525	5.630
N3	3.414	7.474	3.644	3.493	3.533	3.254	3.652	3.971	6.578	3.493	7.281	4.526
N4	3.567	8.805	3.731	3.560	3.278	3.038	3.481	4.041	7.506	3.508	8.386	4.809
N5	3.517	7.759	3.696	3.558	3.594	3.278	3.911	3.982	6.929	3.557	7.759	4.685
N6	3.674	9.155	3.785	3.600	3.558	3.249	3.739	4.011	8.023	3.635	9.012	5.040
N7	3.678	9.276	3.783	3.633	3.592	3.261	3.736	4.110	8.044	3.646	9.053	5.074
N8	3.834	10.614	3.872	3.665	3.526	3.186	3.567	4.067	9.171	3.711	10.338	5.414
N9	3.772	10.125	4.738	4.387	4.727	4.039	2.878	4.405	9.143	4.500	9.643	5.669
N10	3.931	11.553	4.825	4.416	3.462	2.898	2.950	4.368	9.469	4.211	10.740	5.711
N11	3.787	8.678	4.513	4.362	4.300	3.708	3.063	3.858	7.928	4.153	8.685	5.185
N12	3.948	10.130	4.599	4.402	4.330	3.754	2.886	3.936	9.029	4.248	9.979	5.567
N13	3.844	8.990	3.749	3.566	3.494	3.154	3.676	3.816	7.809	3.591	8.747	4.949
N14	3.624	8.496	4.364	4.291	4.113	3.561	3.233	3.668	7.546	3.974	8.492	5.033
N15	3.784	9.786	4.449	4.323	3.239	2.816	3.107	4.069	8.181	3.798	9.347	5.173

*. average predicted activity.

**Tab. 5.** Intercorrelation matrix for Eq. 8 (model A2)

**Descriptor**	**SC-3_P**	**^3^χ_p_**	**S_sCH_3_**	**S_aasC**
**SC-3_P**	1.000	0.989	−0.133	0.020
**^3^χ_p_**		1.000	−0.062	−0.027
**S_sCH_3_**			1.000	−0.566
**S_aasC**				1.000

**Tab. 6.** Results of validation based on randomization.

**Process randomization**						

**Models with topological, structural and thermodynamic descriptors**						

**Model No.**	**Statistical tool**	**R^2^**	**R**	**R_r_**	**R_r_^2^**	**^c^R_p_^2^**

A1	GFA-linear	0.889	0.943	0.531	0.282	0.735
A2	GFA-spline	0.919	0.959	0.533	0.284	0.764
A3	G/PLS-linear	0.856	0.925	0.669	0.448	0.591
A4	G/PLS-spline	0.906	0.952	0.808	0.653	0.479

**Models with spatial, electronic and thermodynamic descriptors**						

**Model No.**	**Statistical tool**	**R^2^**	**R**	**R_r_**	**R_r_^2^**	**^c^R_p_^2^**

B1	GFA-linear	0.824	0.908	0.467	0.218	0.707
B2	G/PLS-linear	0.839	0.916	0.671	0.450	0.571
B3	G/PLS-spline	0.865	0.930	0.778	0.605	0.474

**Models with combined set of descriptors**						

**Model No.**	**Statistical tool**	**R^2^**	**R**	**R_r_**	**R_r_^2^**	**^c^R_p_^2^**

C1	GFA-linear	0.935	0.967	0.646	0.417	0.696
C2	GFA-spline	0.925	0.962	0.715	0.511	0.619
C3	G/PLS-linear	0.883	0.940	0.686	0.471	0.603
C4	G/PLS-spline	0.919	0.959	0.795	0.632	0.514

**Model randomization**						

**Model No.**		**R^2^**	**R**	**R_r_**	**R_r_^2^**	**^c^R_p_^2^**

A2		0.919	0.959	0.379	0.144	0.844
A3		0.856	0.925	0.058	0.003	0.854
A4		0.906	0.952	0.12	0.014	0.899
C1		0.935	0.967	0.368	0.135	0.865
C2		0.925	0.962	0.377	0.142	0.851
C4		0.919	0.959	0.113	0.013	0.913

## Supporting Information

A table with the values of the important descriptors appearing in the described QSAR models is available in the online version (Format: PDF, Size: < 0.2 MB): http://dx.doi.org/10.3797/scipharm.1011-02
