# Supplementary material for: Chemometric QSAR Modeling and In Silico Design of Antioxidant NO Donor Phenols
Source: Sci Pharm. 2010 Dec 2;79(1):31–57. doi: 10.3797/scipharm.1011-02 (PMC3097501; doi:10.3797/scipharm.1011-02)
Supplement: Supplementary file 1 [file Scipharm_2011_79_31_supporting_information.pdf]

Scientia Pharmaceutica www.scipharm.at

Open Access

Supporting Information to

Chemometric QSAR Modeling and *In Silico* Design of Antioxidant NO Donor Phenols

Indrani MITRA, Achintya SAHA, Kunal ROY

Published in Sci Pharm. 2011; 79: 31–57 doi:10.3797/scipharm.1011-02

Available from: http://dx.doi.org/10.3797/scipharm.1011-02

© Mitra, Saha and Roy; licensee Österreichische Apotheker-Verlagsgesellschaft m. b. H., Vienna, Austria.

This is an Open Access article distributed under the terms of the Creative Commons Attribution License (http://creativecommons.org/licenses/by/3.0/), which permits unrestricted use, distribution, and reproduction in any medium, provided the original work is properly cited.

Table of Contents

Tab. S1. Values of the important descriptors appearing in the described QSAR models

Tab. S1. Values of the important descriptors appearing in the described QSAR models

**Sl no.**	**Activ.**	***SC-3_P***	***CHI-1***	$^{3}\chi^{p}$	$^{3}\chi^{v}_{c}$	***S_sCH₃***	***S_aasC***	***S_ddsN***	***MR***	***Jurs-RPCG***	***Jurs-TASA***
**1**	0.538	10	3.788	2.305	0.241	1.986	1.499	0.000	35.954	0.347	228.889
**2**	1.745	20	5.685	3.946	0.340	4.923	1.908	0.000	19.697	0.184	304.037
**3**	2.77	28	7.032	4.807	2.867	14.884	3.757	0.000	33.873	0.233	453.550
**4**	3.77	35	7.333	6.331	1.417	10.180	5.594	0.000	14.472	0.223	405.845
**5**	0.959	36	8.570	6.290	0.878	1.642	−1.009	−0.173	45.130	0.443	352.508
**6**	0.845	17	6.682	3.779	0.223	0.000	1.321	0.000	49.984	0.488	308.958
**7**	2.229	27	8.579	5.452	0.322	2.917	1.530	0.000	33.727	0.370	380.277
**8**	2.699	35	9.926	6.312	2.849	12.502	3.379	0.000	47.903	0.415	523.864
**9**	3.824	45	11.250	8.510	1.241	7.819	5.027	0.000	34.110	0.351	510.788
**10**	0.733	23	8.469	4.759	0.371	0.000	0.857	0.000	55.043	0.304	300.617
**11**	2.268	33	10.367	6.432	0.470	2.727	0.712	0.000	38.786	0.254	396.410
**12**	2.585	41	11.713	7.292	2.997	11.930	2.561	0.000	52.962	0.274	508.129
**13**	1.328	50	12.481	9.203	1.070	0.000	0.079	−0.169	82.521	0.372	455.907
**14**	2.469	60	14.379	10.876	1.170	2.859	0.053	−0.187	66.263	0.323	518.681
**15**	2.699	68	15.725	11.736	3.696	12.326	2.002	−0.150	80.439	0.343	597.705
**16**	3.31	74	15.049	12.947	2.089	7.394	3.042	−0.170	52.007	0.344	567.791
**17**	2.921	55	13.713	9.647	3.273	14.480	3.429	0.046	66.343	0.228	616.445
**18**	3.854	61	13.037	10.709	1.666	9.610	4.724	0.030	37.910	0.229	472.813
**19**	1.77	24	6.271	4.435	0.347	1.862	0.837	0.000	39.255	0.273	333.213
**20**	1.699	28	7.203	5.254	0.415	3.458	1.545	0.000	47.852	0.220	367.604
**21**	2.469	34	10.059	6.570	0.446	1.818	1.360	0.000	61.882	0.378	448.296
**22**	2.678	37	11.559	7.320	0.446	1.839	1.502	0.000	75.429	0.353	518.432
**23**	2.538	43	13.346	8.358	0.594	1.807	1.326	0.000	80.487	0.246	516.217
**24**	2.886	59	14.614	10.752	0.637	1.829	2.428	0.333	77.167	0.227	590.189
**25**	2.42	67	15.858	11.994	1.293	1.799	0.031	−0.188	94.418	0.326	591.734
**26**	2.102	48	11.452	8.586	0.646	1.784	0.651	−0.066	57.153	0.238	366.155
**27**	2.237	46	11.080	8.423	0.587	1.794	0.997	0.029	46.004	0.268	441.425
**28**	0.343	30	8.968	5.961	0.360	0.000	0.000	0.000	36.136	0.271	233.852
**29**	1.097	33	10.468	6.711	0.360	0.000	0.000	0.000	49.682	0.258	320.336
**30**	1.553	39	12.256	7.748	0.509	0.000	0.000	0.000	54.741	0.196	345.805
**31**	1.77	55	13.524	10.142	0.552	0.000	0.710	0.242	59.962	0.173	389.958
**32**	1.097	63	14.768	11.384	1.208	0.000	−1.521	−0.278	68.672	0.269	401.781
**33**	0.407	55	13.524	10.101	0.553	0.000	0.707	0.241	51.421	0.173	377.539

Article published in: Sci Pharm. 2011; 79: 31–57. doi:10.3797/scipharm.1011-02
